# Transcriptomic Profiles in Children With Septic Shock With or Without Immunoparalysis

**DOI:** 10.3389/fimmu.2021.733834

**Published:** 2021-10-01

**Authors:** Andrew Snyder, Kathleen Jedreski, James Fitch, Saranga Wijeratne, Amy Wetzel, Josey Hensley, Margaret Flowers, Katherine Bline, Mark W. Hall, Jennifer A. Muszynski

**Affiliations:** ^1^ Center for Clinical and Translational Research, Abigail Wexner Research Institute at Nationwide Children’s Hospital, Columbus, OH, United States; ^2^ Institute for Genomic Medicine, Abigail Wexner Research Institute at Nationwide Children’s Hospital, Columbus, OH, United States; ^3^ Division of Critical Care Medicine, Nationwide Children’s Hospital, Columbus, OH, United States

**Keywords:** pediatric, sepsis, innate immunity, adaptive immunity, transcriptome

## Abstract

**Background:**

Severe innate immune suppression, termed immunoparalysis, is associated with increased risks of nosocomial infection and mortality in children with septic shock. Currently, immunoparalysis cannot be clinically diagnosed in children, and mechanisms remain unclear. Transcriptomic studies identify subsets of septic children with downregulation of genes within adaptive immune pathways, but assays of immune function have not been performed as part of these studies, and little is known about transcriptomic profiles of children with immunoparalysis.

**Methods:**

We performed a nested case-control study to identify differences in RNA expression patterns between children with septic shock with immunoparalysis (defined as lipopolysaccharide (LPS)-induced tumor necrosis factor (TNF)α response < 200 pg/ml) *vs* those with normal LPS-induced TNFα response. Children were enrolled within 48 hours of the onset of septic shock and divided into two groups based on LPS-induced TNFα response. RNA was extracted from whole blood for RNAseq, differential expression analyses using DESeq2 software, and pathway analyses using Ingenuity Pathway Analysis.

**Results:**

32 children were included in analyses. Comparing those with immunoparalysis (n =19) to those with normal TNFα response (n = 13), 2,303 transcripts were differentially expressed with absolute value fold change ≥ 1.5 and false discovery rate ≤ 0.05. The majority of downregulated pathways in children with immunoparalysis were pathways that involved interactions between innate and adaptive immune cells necessary for cell-mediated immunity, crosstalk between dendritic cells and natural killer cells, and natural killer cell signaling pathways. Upregulated pathways included those involved in humoral immunity (T helper cell type 2), corticotropin signaling, platelet activation (GP6 signaling), and leukocyte migration and extravasation.

**Conclusions:**

Our study suggests that gene expression data might be useful to identify children with immunoparalysis and identifies several key differentially regulated pathways involved in both innate and adaptive immunity. Our ongoing work in this area aims to dissect interactions between innate and adaptive immunity in septic children and to more fully elucidate patient-specific immunologic pathophysiology to guide individualized immunotherapeutic targets.

## Introduction

Septic shock is an important source of morbidity and mortality in children worldwide (1, 2). While the signs and symptoms of sepsis arise from an overwhelming inflammatory response, work from our group and others has demonstrated significant associations between suppressed innate immune response and adverse outcomes in critically ill septic children (3–5). Sepsis-induced innate immune suppression is typically occult but can be identified by quantifying production of tumor necrosis factor (TNF)α, an inflammatory cytokine, in whole blood after stimulation with lipopolysaccharide (LPS). Using this standardized assay, we have previously identified a threshold *ex vivo* LPS-induced TNFα response of < 200 pg/ml, termed immunoparalysis, that is associated with the development of nosocomial infection, prolonged organ dysfunction, and/or increased mortality in multiple studies of children with severe sepsis and/or septic shock (3–5). While the *ex vivo* LPS-induced TNFα assay is highly reproducible, its widespread use is limited by the need for a centralized laboratory for reagent preparation and cytokine quantitation, and on-site sample processing at the time of the blood draw. Alternate methods to identify children with clinically relevant ICU-related immune suppression are needed to facilitate multicenter clinical trial design and translation to the bedside.

Previous transcriptomic studies of children with septic shock have identified associations between downregulation of genes involved in adaptive immunity and adverse clinical outcomes, suggesting that gene expression patterns might be useful to identify children with septic shock with clinically relevant immune suppression (6–8). However, assays of immune cell function were not performed in these studies, and little is known about gene expression profiles in children with sepsis-induced immunoparalysis. We therefore performed a pilot nested case-control study to identify differences in RNA expression patterns between children with septic shock with immunoparalysis *versus* those with normal LPS-induced TNFα response.

## Methods

### Setting

The pediatric intensive care unit (PICU) at Nationwide Children’s Hospital is a 54-bed, quaternary care, medical/surgical intensive care unit that sees approximately 3,000 annual admissions. Patients admitted to a separate cardiothoracic intensive care unit were not included in this study. The study was approved by the Institutional Review Board at Nationwide Children’s Hospital.

### Patients

After informed consent, children who were within 48 hours of septic shock onset were enrolled. Septic shock was defined by the presence of sepsis (per consensus criteria) and the use of continuous vasoactive medication for shock (9). Children were excluded from the parent study if they had a limitation of care order in place or likely progression to brain death (as determined by the treating attending physician). Children who required extracorporeal membrane oxygenation support within 48 hours of septic shock onset were excluded from these analyses because of the likelihood that their transcriptomic profiles would be very different and an insufficient number of patients to permit stratified analyses. To ensure that enough RNA would be available for analysis, children were also excluded if their total white blood cell count was < 1,000 cells/mm^3^.

LPS-induced TNFα production capacity was assessed within 48 hours of septic shock onset as previously described (5). Briefly, patient samples were drawn into heparinized blood tubes (Becton Dickson, Franklin Lakes, NJ) within 48 hours of the onset of septic shock. 50 microliters of whole blood were added to pre-prepared stimulation solution containing RPMI and 0.5 ng/ml LPS (phenol-extracted from Salmonella abortus equi [Sigma, St. Louis, MO]) and incubated for four hours at 37°C. After four hours, the supernatant was collected and stored at -80°C for batch analysis of TNFα, which was quantified by chemiluminescence using the *Immulite 1000* automated chemiluminometer (Siemens Healthcare Diagnostics, Deerfield, Il). Stimulation assays were performed in duplicate and values reported are the average values from each set of duplicates.

To ensure adequate separation between groups, children were selected and divided into two groups based on their LPS-induced TNFα response as follows: Immunoparalysis (LPS-induced TNFα response < 200 pg/ml) and normal innate immune function (LPS-induced TNFα response > 830 pg/ml). The definition of immunoparalysis is derived from our previous publications (3–5, 10, 11). The threshold of 830 pg/ml represents the 25%ile of LPS-induced TNFα response in healthy children (5). Children with an initial LPS-induced TNFα response between 200 and 830 pg/ml were not included in these analyses. As we were interested in stable immune phenotypes, children whose LPS-induced TNFα response significantly changed over the course of 48 hours (i.e. transient immunoparalysis or transient normal) were also not included.

### Clinical Data Collection and Definitions

Clinical data was abstracted from the electronic medical record into a standardized, electronic case report form by trained research coordinators. Complex chronic conditions were defined as previously published (12). Severity of illness was quantified by the pediatric risk of mortality (PRISMIII) score on the day of septic shock onset (13). Vasoactive inotrope score was used to define intensity of treatment for shock, as previously published (14). Nosocomial sepsis was defined as the onset of septic shock after 48 hours of hospital admission.

### RNA Extraction

Blood samples for RNA extraction were drawn at the same time as samples drawn for LPS-induced TNFα production capacity assays. Whole blood (0.6 to 1ml) was drawn into tubes containing 1 ml of Tempus™ RNA stabilization solution (Applied Biosystems). The tubes were shaken vigorously then immediately frozen and stored at -20°C for batch analysis. RNA was isolated using the MagMax for Stabilized Blood Tubes RNA Isolation kit (Life Technologies) *via* a modification of the manufacture’s protocol to permit small volume blood sampling as follows (15). 100 ul of Tempus 1x PBS was added to each blood sample and vortexed for 30 seconds. Tubes were centrifuged at 4,000 x g for 20 minutes at 4°C to pellet the crude RNA. Pellets were washed with 4ml Tempus Pre-Digestion Wash, centrifuged at 4,000 x g, resuspended in 120 ul of Tempus Resuspension Solution and Proteinase and vortexed for 10 seconds to resuspend the pellet. 10 ul of TURBO™ DNase was added to the tubes and vortexed for 10 minutes. 50 ul of Binding Solution Concentrate and 20 ul of RNA binding beads were then added and the tube was vortexed for 1 minute. 200 ul of 100% isopropanol was added for another 3-minute vortex. A magnetic stand was used to capture the RNA binding beads with the supernatant discarded. 150 ul of Wash Solution 1 was added to the tube and vortexed for 1 minute. The tube was then placed on the magnetic stand to capture the RNA Binding beads, discarding the supernatant. This wash was repeated once. 150 ul of Wash Solution 2 was added to the tube and vortexed for 1 minute then put back on the magnetic stand. The supernatant was carefully discarded, and the wash was repeated. The tubes were inverted on absorbent paper for 2 minutes to dry the beads then 60 ul of Elution Buffer was added. The tubes were vortexed for 4 minutes and put back on the magnetic stand. The RNA-containing supernatant was extracted from the tubes and stored at -20°C.

### RNA Sequencing

Strand-specific RNA-seq libraries were prepared using NEBNext Ultra II Directional RNA Library Prep Kit for Illumina, following the manufacturer’s recommendations. In summary, total RNA quality was assessed using RNA 6000 Nano kit on Agilent 2100 Bioanalyzer (Agilent Biotechnologies) and concentration measured using Qubit RNA HS assay kit (Life Technologies). An aliquot of total RNA was rRNA depleted using NEB’s Human/Mouse/Rat RNAse-H based Depletion kit (New England BioLabs). Following rRNA removal, mRNA was fragmented and then used for first- and second-strand cDNA synthesis with random hexamer primers. ds cDNA fragments underwent end-repair and a-tailing and ligated to dual-unique adapters (Integrated DNA Technologies). Adaptor-ligated cDNA was amplified by limit-cycle PCR. Library quality was analyzed on Tapestation High-Sensitivity D1000 ScreenTape (Agilent Biotechnologies) and quantified by KAPA qPCR (KAPA BioSystems). Libraries were pooled and sequenced at 2 x 150 bp read lengths to generate approximately 60-80 million paired-end reads per sample.

### Statistical Analyses

Clinical characteristics were compared using Fisher’s exact test (categorical variables) or Wilcoxon rank sum test (continuous variables), as appropriate, using Prism 9 (Graph Pad, San Diego, CA). A p-value of < 0.05 was considered significant for these analyses.

### RNAseq Data Processing and Differential Expression Analysis

Low-quality reads (q<10) and adaptor sequences were eliminated from raw reads using bbduk version 37.64 (16). Each sample was aligned to the GRCh38.p9 assembly of the Homo Sapiens reference from NCBI (17) using version 2.6.0c of the RNA-Seq aligner STAR (18). Features were identified from Gencode (V28). Feature coverage counts were calculated using feature Counts (19). Normalization and differential expression analysis was performed with DESeq2 as previously described (20). No biases were apparent on visual inspection of PCA plots and therefore no batch correction was applied. Significant differentially expressed genes between the two groups are those that have an absolute value of fold change ≥ 1.5 and a false discovery rate (adjusted p-value) of < 0.05 (5% FDR). To visualize the data, a volcano plot was constructed by plotting the log2 fold change by the negative log adjusted p-value for each transcript. Focusing on the top 50 differentially expressed transcripts (sorted by absolute value of fold change) a heatmap was generated by hierarchical clustering using the pheatmap R package version 1.0.12.

### Pathway Analysis

For pathway analyses, the dataset was uploaded to Ingenuity Pathway Analysis (QIAGEN Inc., https://www.quiagenbioinformatics.com/products/ingenuity-pathway-analysis) and analyzed using core analysis within the software with the log ratio cutoffs set to -1.5 to 1.5 and the false discovery rate set to 0.05. These thresholds resulted in 254 differentially expressed transcripts analyzed (123 upregulated and 131 downregulated).

## Results

### Patient Characteristics

A total of 68 patients were enrolled in the parent study. For the purposes of these analyses, 19 patients with immunoparalysis and 13 patients with normal LPS-induced TNFα production capacity were included (**Supplementary Figure 1**). Patient demographics are in **Table 1**. All patients were treated with vasoactive support, with a median vasoactive-inotrope score of 11.5. Overall, 84% required invasive mechanical ventilation. Mortality was 3%, and ICU length of stay was a median of 7.6 [4.2, 12.8] days. Half of patients had a complex chronic condition, most commonly developmental delay and/or seizure disorder (n = 5), chronic lung disease and/or tracheostomy (n = 2), chronic kidney disease (n = 2), and inflammatory bowel disease (n = 2). 16% of patients were immune compromised at baseline due to immunosuppressive medications, including two patients who were status post hematopoietic stem cell transplant (both in the non-immunoparalysis group). Patients with immunoparalysis had higher severities of illness and were less likely to have a complex chronic condition or gram-negative infection. As expected, based on our study design, median *ex vivo* TNFα response for patients with immunoparalysis was 128 [78, 168] pg/ml *vs.* 1293 [1115, 2025] for those with normal TNFα response.

**Table 1:** Patient Characteristics.

Characteristic	All Patients (n = 32)	Immunoparalysis (n = 19)	No Immunoparalysis (n = 13)	p^a^
Male Sex, n (%)	12 (38)	8 (42)	4 (31)	0.7
Age, years	9.4 [2.6, 15.3]	9.3 [2.8, 15.3]	10.3 [2.2, 15]	0.9
Complex Chronic Condition, n (%)	16 (50)	6 (32)	10 (77)	0.03
Immune compromise dx	5 (16)	2 (11)	3 (23)	0.4
PRISM III	12 [9, 17]	16 [10, 21]	10 [5.5, 13]	0.03
Vasoactive Inotrope Score	11.5 [8, 26]	18 [10, 30]	9 [5.5, 20]	0.1
Invasive Mechanical Ventilation, (%)	27 (84)	16 (84)	11 (85)	1
Community-acquired infection, n (%)	31 (97)	19 (100)	12 (92)	0.4
Infection source, n (%)^b^				
Blood	8 (25)	5 (26)	3 (23)	1
Lung	12 (38)	8 (42)	4 (31)	0.7
Urine	4 (13)	1 (5)	3 (23)	0.3
Abdomen	6 (19)	5 (26)	1 (8)	0.4
Wound/soft tissue/bone	1 (3)	1 (5)	0	1
Culture negative/unknown	2 (6)	1 (5)	1 (8)	1
Organism, n (%)^b^				
Gram +	12 (38)	9 (47)	3 (23)	0.3
Gram -	6 (19)	1 (5)	5 (38)	0.03
Polymicrobial	3 (9)	3 (16)	0	0.3
Viral	6 (19)	3 (16)	3 (23)	0.7
Fungal	2 (6)	2 (10)	0	0.5
Absolute neutrophil count, cells/µL	7941 [4134, 12,243]	6960 [4134, 11844]	8365 [5346, 15316]	0.6
Absolute lymphocyte count, cells/µL	1192 [770, 2314]	1026 [565, 1382]	1363 [1130, 2733]	0.1
Absolute monocyte count, cells/µL	767 [351, 1470]	342 [106, 740]	1035 [767, 1637]	0.001

a p-value for immunoparalysis vs. no immunoparalysis.

b Some patients with more than one source and/or more than one organism. PRISM, pediatric risk of mortality. Data are median [interquartile range] except where otherwise specified.

### Differentially Expressed Transcripts

**Figure 1:**
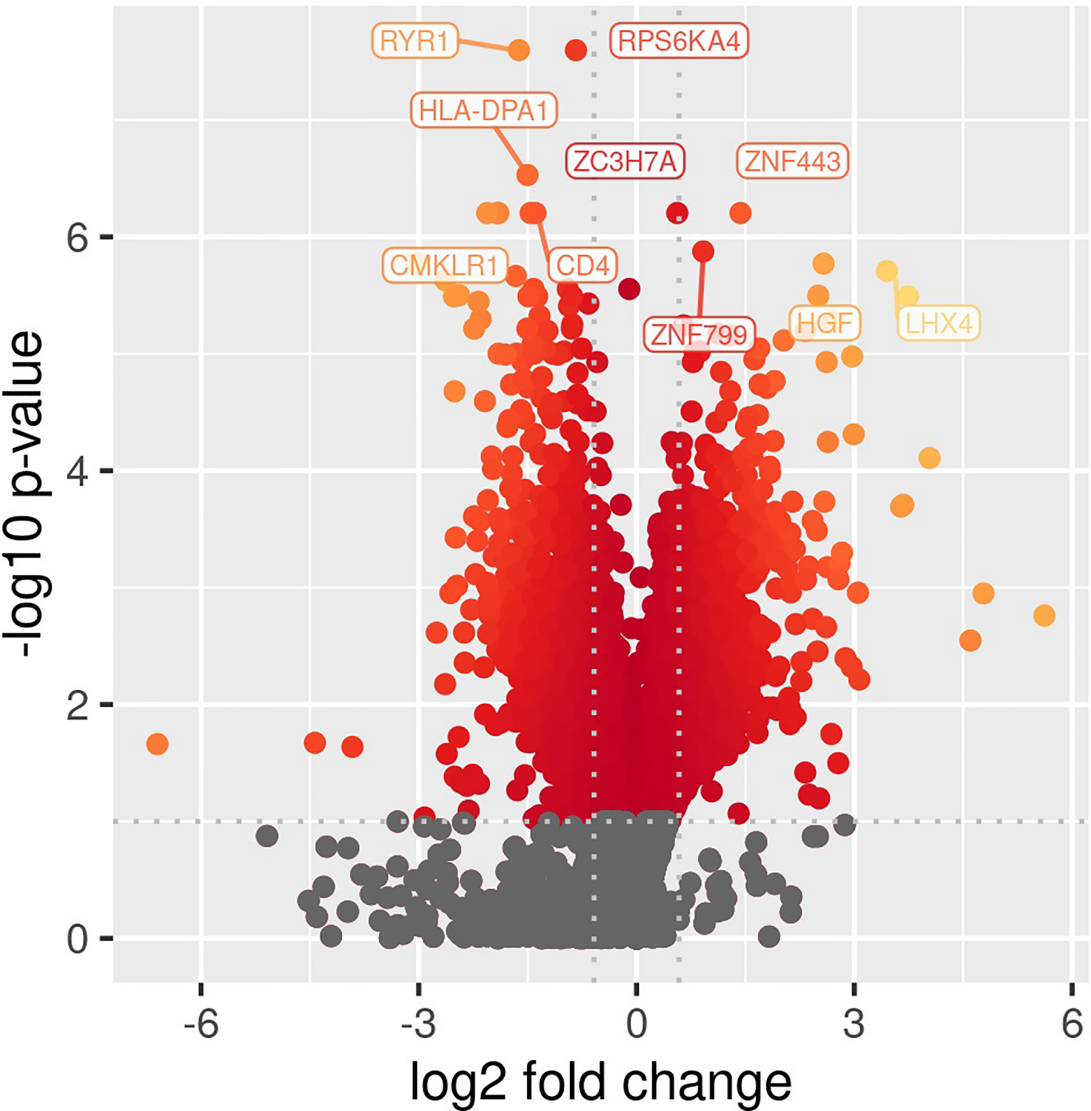
Volcano plot of differentially expressed transcripts comparing children with septic shock and immunoparalysis *versus* children with septic shock and normal *ex vivo* LPS-induced TNFα response (2,303 transcripts). Color represents significant changes in expression, with yellow color indicating greater changes compared to red color. Log fold change is depicted on the X-axis and -log(p value) is on the y axis.

**Figure 2:**
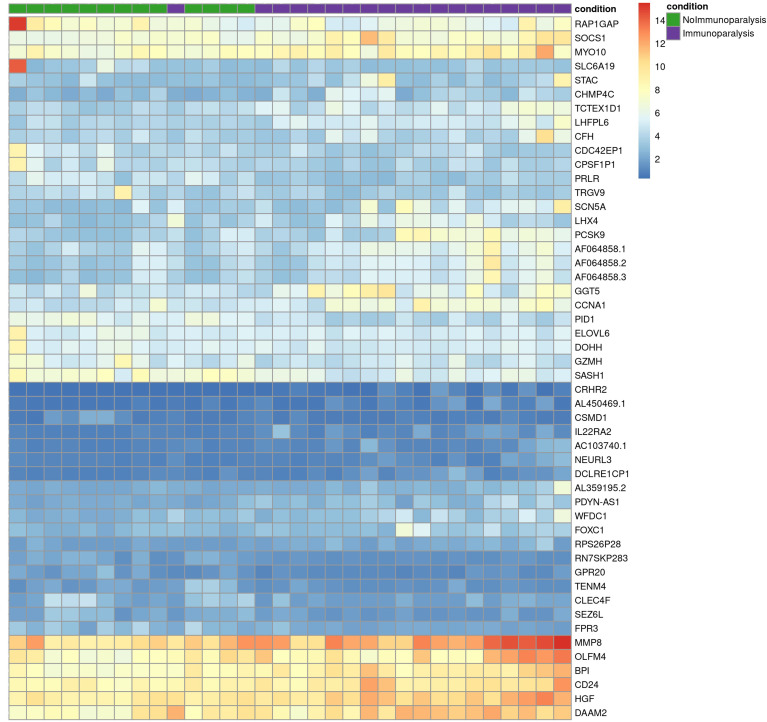
Heat map of the top 50 differentially expressed transcripts comparing children with septic shock and immunoparalysis (purple color) *versus* children with septic shock and normal *ex vivo* LPS-induced TNFα response (green color). Each vertical column represents an individual patient and each row represents an individual transcript. The spectrum of red to blue color represents the Z-score (relative expression) for each transcript from high (red) to low (blue).

Comparing patients with immunoparalysis to those with normal LPS-induced TNFα production capacity, 2,303 transcripts were differentially expressed (**Figure 1**). A heat map generated by hierarchical cluster analysis of the top 50 differentially expressed transcripts between groups is displayed in **Figure 2**. A complete list of differentially expressed transcripts is in **Supplementary Table 1**.

### Pathway Analyses

**Figure 3:**
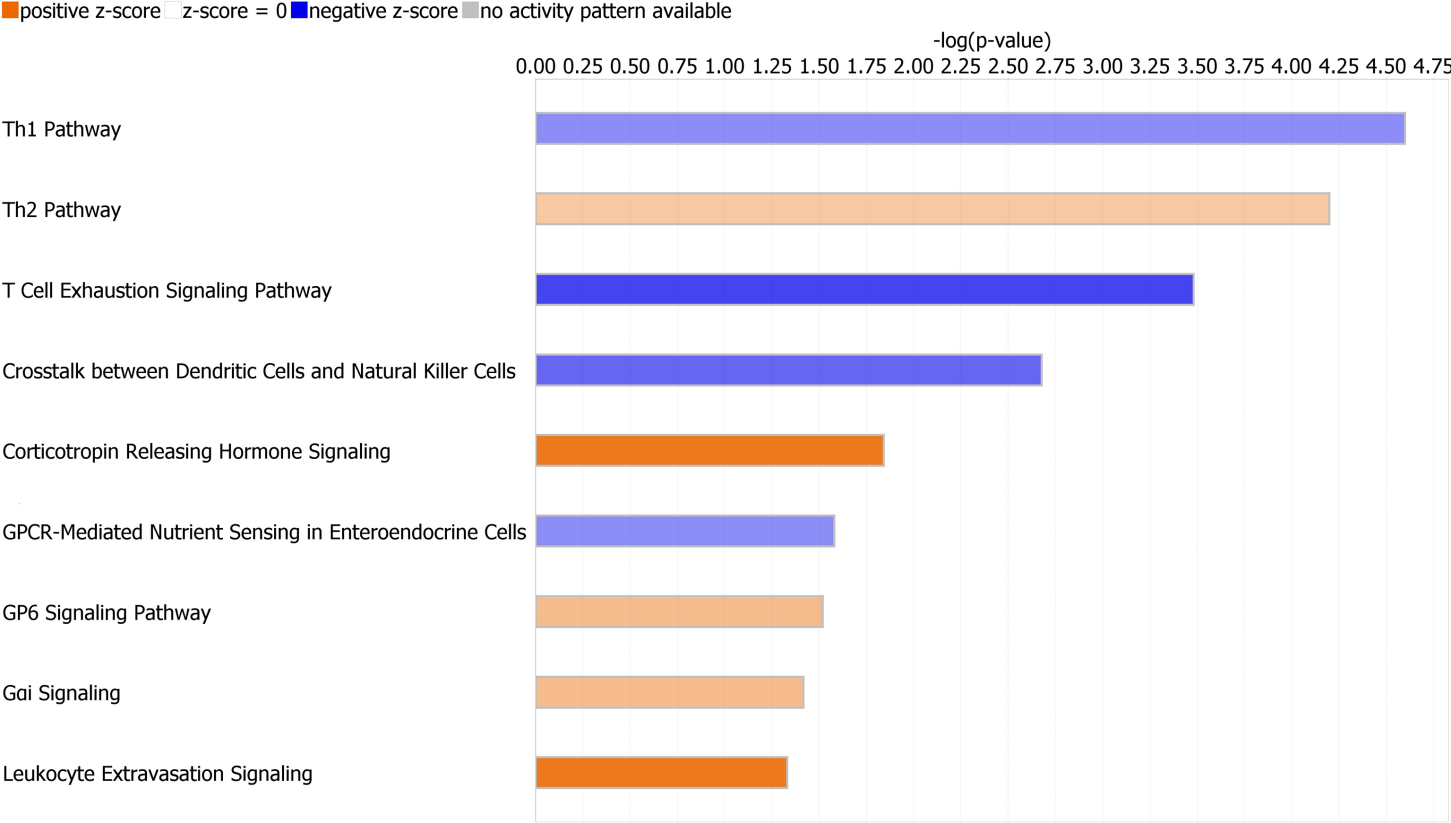
Graph depicting canonical pathways identified by Ingenuity Pathway Analysis (Qiagen). Pathways displayed are those with an absolute value Z-score > 0.5 and -log (p value) > 1.3 by Fisher’s exact test. Upregulated pathways are in orange, and downregulated pathways are in blue. Darker shading indicates greater absolute value Z-scores.

Canonical pathways identified by Ingenuity Pathway Analysis are in **Table 2** and **Figure 3**. The majority of downregulated pathways in children with immunoparalysis were pathways involved in interactions between innate and adaptive immune cells necessary for cell-mediated immunity including T helper cell type 1, T cell exhaustion, crosstalk between dendritic cells and natural killer cells, and natural killer cell signaling pathways. Upregulated pathways included those involved in humoral immunity (T helper cell type 2), corticotropin signaling, platelet activation (GP6 signaling), and leukocyte migration and extravasation. Figures depicting the top 4 canonical pathways (ordered by -log p-value) are in **Supplementary Figures 2–5**.

**Table 2:** Differentially regulated pathways.

Canonical Signaling Pathway	-log(p-value)	Differentially expressed molecules in the pathway
**Downregulated Pathways**		
Th1 pathway	4.6	*CCR5, HLA-DPA1, HLA-DQA2, IL12RB2, IL18R1, KLRC1, SOCS1, TBX21*
T cell exhaustion pathway	3.5	*EOMES, HLA-DPA1, HLA-DQA2, IL12RB2, PPP2R2B, TBX21, TGFBR3, TRGV9*
Crosstalk between dendritic cells and natural killer cells	2.7	*CD209, FASLG, IL2RB, IL3RA, PRF1*
GPCR-mediated nutrient sensing in enteroendocrine cells	1.6	*ADCY6, CACNA2D3, FFAR3, FFAR4*
Synaptic long term depression	1.4	*CACNA2D3, GUCY1B1, PLA2G7, PPP2R2B, RYR1*
Natural killer cell signaling	1.4	*FASLG, IL12RB2, IL18R1, IL2RB, KLRC1*
**Upregulated Pathways**		
Th2 pathway	4.2	*CCR5, CXCR6, HLA-DPA1, HLA-DQA2, IL12RB2, IL2RB, TBX21, TGFBR3*
Corticotropin releasing hormone signaling	1.8	*ADCY6, CACNA2D3, CRHR2, FASLG, GUCY1B1*
GP6 signaling pathway	1.5	*COL17A1, ITGA2B, ITGB3, LAMA2*
Gαi signaling	1.4	*ADCY6, FFAR3, RAP1GAP, XCR1*
Leukocyte extravasation pathway	1.3	*CLDN23, ITGB3, MMP27, MMP8, RAP1GAP*

Th1, T helper type 1 cells; CCR5, chemokine receptor type 5; HLA-DPA1, major histocompatibility complex class II; DP, alpha 1; HLA-DQA2, major histocompatibility complex class II, DQ, alpha 2; IL12RB2, interleukin 12 receptor subunit beta 2; IL18R1, interleukin 18 receptor 1; KLRC1, killer cell lectin-like receptor C1; SOCS1, suppressor of cytokine signaling 1; TBX21, T-box transcription factor 21 (aka T-cell-specific T-Box factor T-Bet); EOMES, eomesodermin; PPP2R2B, protein phosphatase 2 regulatory subunit B beta; TGFBR3, transforming growth factor beta receptor 3; TRGV9, T cell receptor gamma variable 9; FASLG, Fas Ligand; IL2RB, interleukin 2 receptor subunit beta; IL3RA, interleukin 3 receptor subunit alpha; PRF1, perforin 1; GPCR, G protein coupled receptor; ADCY6, adenylate cyclase 6; CACNA2D3, calcium voltage-gated channel auxiliary subunit alpha2/delta3; FFAR3, free fatty acid receptor 3; free fatty acid receptor 4; GUCY1B1, guanylate cyclase 1 soluble subunit beta 1; PLA2G7, phospholipase A2 group VII; RYR1, ryanodine receptor 1; Th2, T helper type 2 cells; CXCR6, C-X-C motif chemokine receptor 6; CRHR2, corticotropin releasing hormone receptor 2; GP6, platelet glycoprotein VI; COL17A1, collagen type XVII alpha 1 chain; ITGA2B, integrin subunit alpha 2b; ITGB3, integrin subunit beta 3; LAMA2, laminin subunit alpha 2; Gαi, G protein alpha-subunit I; RAP1GAP, rap1 GTPase-activating protein; XCR1, X-C motif chemokine receptor 1; CLDN23, claudin 23; MMP27, matrix metalloproteinase 27; MMP8, matrix metalloproteinase 8.

## Discussion

Our results support the hypothesis that transcriptome profiling might identify critically ill septic children with immunoparalysis and provide hypothesis-generating data to inform potential mechanisms of sepsis-induced immune suppression in critically ill children. In our cohort, immunoparalysis was associated with downregulation of pathways involved in antigen presentation by innate immune cells, cell-mediated effector T-cell responses, and natural killer cell function. Pathways involved in platelet activation, leukocyte migration, and corticotropin releasing hormone signaling were upregulated. These data suggest that mechanisms of sepsis-induced immunoparalysis likely involve complex interactions among multiple arms of the immune response.

Innate immune cells play a critical role in propagating and regulating adaptive immune responses. Thus, it is not surprising that children with immunoparalysis display downregulation of pathways involved in interactions between innate and adaptive immune cells and cell-mediated immunity. Skewing of helper T cell activity away from cell mediated (Th1) immunity and toward more immune suppressive (Th2) responses has long been recognized in adult sepsis (21–23). Our previous studies similarly implicate adaptive immune suppression in critically ill septic children (5, 24, 25). In previous transcriptomic analyses of children with septic shock, pathways involved in antigen presentation and T cell receptor signaling have been associated with adverse clinical outcomes, though assays of immune cell function were not performed in these studies (6, 8). Ours is among the first studies to demonstrate an association between innate immune suppression, as assessed by stimulated cytokine production, and downregulation of genes involved in antigen presentation and cell mediated immunity.

Major histocompatibility complex (MHC) class II molecules expressed on innate immune cells play a key role in antigen presentation and activation of effector T cells. Multiple MHC class II molecules were downregulated in children with immunoparalysis in our cohort and contributed to pathway analyses that identified T cell signaling pathways. Such downregulation of MHC class II molecules would be consistent with suppression of innate immune cell function in sepsis. For instance, low surface expression of the MHC class II molecule, HLA-DR, on the surface of circulating monocytes has been shown to predict mortality and/or the development of nosocomial infection in septic adults, though its utility in defining clinically relevant immune suppression in critically ill children has been less well studied (26, 27).

Specific to T cell function, multiple T-cell receptor subunits and the transcription factor, T-bet, were downregulated in children with immunoparalysis. The selective expression of T-bet is essential in Th1 development, and previous studies have shown that T-bet deficient CD4+ T cells fail to trigger an effective Th1 response during bacterial infections (28–31). CD8+ T-cells lacking T-bet have also been shown previously to have defects in their effector functions (32). CD8+ T-cells lacking both T-bet and EOMES, which is also downregulated in children with immunoparalysis in our dataset, lose their cytotoxicity (33). EOMES can substitute for T-bet under some circumstances, and together they act through STAT5 to regulate perforin expression in CD8+ T-cells (34, 35). The under expression of both T-bet and EOMES may provide some explanation for the downregulation of perforin that was also observed in children with immunoparalysis in our cohort. T-bet and EOMES also cooperate to regulate the expression of CD122 to promote cell survival and proliferation of memory CD8+ T-cells (36). Loss of this cooperative regulation may also help explain the observed downregulation of CD122 in our cohort.

Two pathways involved in natural killer cell function were differentially regulated in our cohort. One of the most down-regulated genes in children with immunoparalysis was GZMH, a cytotoxic granule protein expressed by natural killer cells (37–41). Natural killer cells also require proper expression of EOMES and T-bet for normal terminal differentiation, function, and survival (42–44). Alterations in natural killer cell function have been implicated in hyperinflammatory sepsis syndromes that resemble macrophage activation syndrome (MAS) in both adults and children (3, 10, 45). In a recent multicenter observational study of critically ill children with sepsis-induced organ dysfunction, 18% of children with immunoparalysis also met diagnostic criteria for MAS-like sepsis (3). Though not evaluated in the current study, it is possible that alterations in natural killer cell gene expression and/or function may underly a subset of children with both immunoparalysis and hyperinflammatory MAS-like sepsis induced organ dysfunction. We view this as an important area of ongoing research.

Two pathways, corticotropin releasing hormone signaling and GPCR-mediated nutrient sensing in enteroendocrine cells, imply disruptions of endocrine signaling in children with immunoparalysis. The most up-regulated protein-coding gene in our dataset was corticotropin releasing hormone receptor 2 (CRHR2). Corticotropin releasing hormone is chiefly responsible for regulating the hypothalamic-pituitary-adrenal axis. Immune cells have been shown to express CRHR2, with differential effects (46, 47). For instance, CRHR2 inhibition was shown to decrease neutrophil-mediated inflammation in a murine stress-pneumonia model (48). Conversely, stimulation of CRHR2 on splenic B cells led to an increase in B cell apoptosis (49). These findings mirror our findings of upregulation of neutrophil activation along with downregulation of lymphocyte signaling pathways and supports the hypothesis that immunoparalysis likely represents a mixed state of immune dysregulation characterized by simultaneous systemic inflammation and functional immune suppression. Gαi signaling, anther G protein signaling pathway, was also upregulated in children with immunoparalysis. G-protein singling is involved in many aspects of cellular function, including immune cell function. Specifically, Gαi has previously been implicated in acute inflammatory responses, again suggesting concomitant patterns of acute inflammation and immune suppression (50).

Both leukocyte extravasation and platelet activation pathways were upregulated in children with immunoparalysis, highlighting the potential importance of non-immune cell types, e.g. endothelial cells and platelets, to inflammation in children with sepsis-induced immunoparalysis. Claudin proteins play important roles in endothelial and epithelial cell tight junction function, with claudin regulation both aiding leukocyte extravasation to sites of infection and contributing to capillary leak in septic shock (51). Their roles in immunoparalysis are uncertain. As discussed above, immunoparalysis is often seen concurrently with severe inflammation. It is possible that disruption in tight junction function drives further inflammation by increasing capillary leak leading to worsened shock and/or by disrupting intestinal barrier function. We view this as an important hypothesis for future research. Along similar lines, matrix metalloproteinases have been shown to contribute to neutrophil chemotaxis and migration and to neutrophil antimicrobial activity (phagocytosis and neutrophil extracellular trap formation) (52). While previous studies identified elevated serum concentrations and/or gene expression of MMP-8 in septic non-survivors compared to survivors (53), juvenile animal studies suggest that MMP-8 serves important roles in bacterial clearance, with increased mortality in MMP-8 null mice (52). These studies suggest that MMP-8 elevations seen in septic patients may be reflective of a necessary inflammatory response to clear infection. Again, our findings underscore the importance of understanding mechanisms of concurrent inflammation and immune suppression and of understanding interactions between endothelial cell and neutrophil regulation in pediatric sepsis. Integrins play important roles in both leukocyte and platelet trafficking, adhesion, and activation. ITGB3 binds fibrinogen, serving as a nidus for platelet/platelet interactions, and may be an important link between inflammation and coagulation. Similarly, upregulation of the platelet glycoprotein VI pathway among children with immunoparalysis points to a potential role for platelets in dysregulated immune responses to sepsis. This is in keeping with a growing body of literature suggesting that platelets play important roles in the innate immune response, though little is known about relationships between platelet and immune function in septic children (54).

Our study has limitations. Because of our sample size, we are unable to account for differences in baseline characteristics, including the presence of complex chronic conditions, between groups or to determine the extent to which these differences may have influenced our results. Future, larger studies are needed to identify changes in gene expression associated with immunoparalysis independent of other clinical characteristics. Second, differences in absolute numbers of circulating leukocytes could also influence our results. Specifically, children with immunoparalysis had lower absolute monocyte counts compared to children without immunoparalysis, which could explain downregulation in gene expression pathways associated with antigen presentation. Our ongoing work in this area aims to determine the relative contribution of differential cell counts to the diagnosis of immunoparalysis and its related outcomes and to identify cell type-specific changes in gene expression associated with sepsis-induced inflammation and immune suppression in children. Third, our cohort contains 16% of children with baseline immune compromise and it is possible that RNA expression patterns were influenced by underlying rather than sepsis-induced immune suppression. However, these subjects were evenly divided between immunoparalysis and non-immunoparalysis groups and subjects with underlying immune compromise diagnoses have historically been included in studies of sepsis-induced immunoparalysis and outcomes (3–5). Future studies are needed to ascribe differences in transcriptomic signatures between endogenous and exogenously produced immunoparalysis in children. It should be noted that because we designed our study to evaluate children with severe innate immune suppression/immunoparalysis *versus* those with normal LPS-induced TNFα production capacity, we are unable to comment on potential changes in gene expression along the spectrum of TNFα production capacity values. Lastly, our current study was focused on identifying transcriptomic signatures associated with immunoparalysis in the first 48 hours of septic shock, thus we are not able to comment on changes over time. Immune dysregulation in septic shock can by highly dynamic and understanding the trajectory of immune function and associated transcriptomic changes remains an important future research goal. In conclusion, our study provides proof of principle that gene expression data might be useful to identify children with immunoparalysis and identifies several key differentially regulated pathways involved in both innate and adaptive immunity. Our ongoing work in this area aims at dissecting interactions between innate and adaptive immunity in children with septic shock, establishing patterns of both immune function and transcriptome expression over time, and more fulling elucidating patient-specific immunologic pathophysiology, including the use of machine learning algorithms to predict immunologic phenotype and clinical outcomes and to guide individualized immunotherapeutic targets.

## Data Availability Statement

The datasets presented in this study can be found in online repositories. The names of the repository/repositories and accession number(s) can be found below: ArrayExpress (https://www.ebi.ac.uk/arrayexpress/), E-MTAB-10938.

## Ethics Statement

The studies involving human participants were reviewed and approved by Nationwide Children’s Hospital Institutional Review Board. Written informed consent to participate in this study was provided by the participants’ legal guardian/next of kin.

## Author Contributions

AS and JM conceived of and planned the study, supervised all of the analyses, interpreted the data, and wrote the manuscript. KJ, JH, and MF processed patient samples and collected clinical data. JF, SW, and AW performed and supervised RNA sequencing and data analysis. KB and MH helped plan the study and interpreted the data. All authors contributed to the planning of the study, data acquisition and interpretation. All authors reviewed the manuscript for critical content, and all authors approved the final version of the manuscript submitted for publication. All authors contributed to the article and approved the submitted version.

## Funding

This work was supported by the Nationwide Foundation Pediatric Innovation Fund (Institute of Genomic Services Core), P30CA016058 from the National Cancer Institute (Institute of Genomic Services Core), and K08HL123925 from the National Heart, Lung and Blood Institute (JM).

## Conflict of Interest

The authors declare that the research was conducted in the absence of any commercial or financial relationships that could be construed as a potential conflict of interest.

## Publisher’s Note

All claims expressed in this article are solely those of the authors and do not necessarily represent those of their affiliated organizations, or those of the publisher, the editors and the reviewers. Any product that may be evaluated in this article, or claim that may be made by its manufacturer, is not guaranteed or endorsed by the publisher.

## Supplementary Material

Supplementary Figure 1 Consort diagram depicting patient inclusion and exclusion from analyses and allocation into groups. *Immunoparalysis is defined as *ex vivo* LPS-induced TNFα response < 200 pg/ml, per our previous publications.

Supplementary Figure 2 Figure depicting molecules in the Th1 Pathway. Green color represents molecules that are downregulated in the IPA dataset. Red color represents molecules that are upregulated in the IPA dataset. Blue color represents molecules that are predicted to be inhibited based on the IPA dataset. Orange color represents molecules that are predicted to be activated based on the IPA dataset.

Supplementary Figure 3 Figure depicting molecules in the Th2 Pathway. Green color represents molecules that are downregulated in the IPA dataset. Red color represents molecules that are upregulated in the IPA dataset. Blue color represents molecules that are predicted to be inhibited based on the IPA dataset. Orange color represents molecules that are predicted to be activated based on the IPA dataset.

Supplementary Figure 4 Figure depicting molecules in the T cell Exhaustion Pathway. Green color represents molecules that are downregulated in the IPA dataset. Red color represents molecules that are upregulated in the IPA dataset. Blue color represents molecules that are predicted to be inhibited based on the IPA dataset. Orange color represents molecules that are predicted to be activated based on the IPA dataset.

Supplementary Figure 5 Figure depicting molecules in the Crosstalk between Dendritic Cells and Natural Killer Cells Pathway. Green color represents molecules that are downregulated in the IPA dataset. Red color represents molecules that are upregulated in the IPA dataset. Blue color represents molecules that are predicted to be inhibited based on the IPA dataset. Orange color represents molecules that are predicted to be activated based on the IPA dataset.

## References

[1] Rudd KE Johnson SC Agesa KM Shackelford KA Tsoi D Kievlan DR. Global, Regional, and National Sepsis Incidence and Mortality, 1990-2017: Analysis for the Global Burden of Disease Study. Lancet (2020) 395(10219):200–11. doi: 10.1016/S0140-6736(19)32989-7 PMC6970225 31954465

[2] Weiss SL Fitzgerald JC Pappachan J Wheeler D Jaramillo-Bustamante JC Salloo A. Global Epidemiology of Pediatric Severe Sepsis: The Sepsis Prevalence, Outcomes, and Therapies Study. Am J Respir Crit Care Med (2015) 191(10):1147–57. doi: 10.1164/rccm.201412-2323OC PMC4451622 25734408

[3] Carcillo JA Berg RA Wessel D Pollack M Meert K Hall M. A Multicenter Network Assessment of Three Inflammation Phenotypes in Pediatric Sepsis-Induced Multiple Organ Failure. Pediatr Crit Care Med (2019) 20(12):1137–46. doi: 10.1097/PCC.0000000000002105 PMC8121153 31568246

[4] Hall MW Knatz NL Vetterly C Tomarello S Wewers MD Volk HD. Immunoparalysis and Nosocomial Infection in Children With Multiple Organ Dysfunction Syndrome. Intensive Care Med (2011) 37(3):525–32. doi: 10.1007/s00134-010-2088-x PMC5224706 21153402

[5] Muszynski JA Nofziger R Moore-Clingenpeel M Greathouse K Anglim L Steele L. Early Immune Function and Duration of Organ Dysfunction in Critically III Children With Sepsis. Am J Respir Crit Care Med (2018) 198(3):361–9. doi: 10.1164/rccm.201710-2006OC PMC6835060 29470918

[6] Wong HR. Genome-Wide Expression Profiling in Pediatric Septic Shock. Pediatr Res (2013) 73(4 Pt 2):564–9. doi: 10.1038/pr.2013.11 PMC3615026 23329198

[7] Wong HR Cvijanovich NZ Allen GL Thomas NJ Freishtat RJ Anas N. Validation of a Gene Expression-Based Subclassification Strategy for Pediatric Septic Shock. Crit Care Med (2011) 39(11):2511–7. doi: 10.1097/CCM.0b013e3182257675 PMC3196776 21705885

[8] Wong HR Cvijanovich NZ Anas N Allen GL Thomas NJ Bigham MT. Developing a Clinically Feasible Personalized Medicine Approach to Pediatric Septic Shock. Am J Respir Crit Care Med (2015) 191(3):309–15. doi: 10.1164/rccm.201410-1864OC PMC4351580 25489881

[9] Goldstein B Giroir B Randolph A. International Pediatric Sepsis Consensus Conference: Definitions for Sepsis and Organ Dysfunction in Pediatrics. Pediatr Crit Care Med J Soc Crit Care Med World Fed Pediatr Intensive Crit Care Societies (2005) 6(1):2–8. doi: 10.1097/01.PCC.0000149131.72248.E6 15636651

[10] Carcillo JA Halstead ES Hall MW Nguyen TC Reeder R Aneja R. Three Hypothetical Inflammation Pathobiology Phenotypes and Pediatric Sepsis-Induced Multiple Organ Failure Outcome. Pediatr Crit Care Med J Soc Crit Care Med World Fed Pediatr Intensive Crit Care Societies (2017) 18(6):513–23. doi: 10.1097/PCC.0000000000001122 PMC5457354 28410274

[11] Hall MW Geyer SM Guo CY Panoskaltsis-Mortari A Jouvet P Ferdinands J. Innate Immune Function and Mortality in Critically Ill Children With Influenza: A Multicenter Study. Crit Care Med (2013) 41(1):224–36. doi: 10.1097/CCM.0b013e318267633c PMC3705720 23222256

[12] Feudtner C Feinstein JA Zhong W Hall M Dai D. Pediatric Complex Chronic Conditions Classification System Version 2: Updated for ICD-10 and Complex Medical Technology Dependence and Transplantation. BMC Pediatr (2014) 14:199. doi: 10.1186/1471-2431-14-199 25102958 PMC4134331

[13] Pollack MM Patel KM Ruttimann UE. PRISM III: An Updated Pediatric Risk of Mortality Score. Crit Care Med (1996) 24(5):743–52. doi: 10.1097/00003246-199605000-00004 8706448

[14] Gaies MG Gurney JG Yen AH Napoli ML Gajarski RJ Ohye RG. Vasoactive-Inotropic Score as a Predictor of Morbidity and Mortality in Infants After Cardiopulmonary Bypass. Pediatr Crit Care Med J Soc Crit Care Med World Fed Pediatr Intensive Crit Care Societies (2010) 11(2):234–8. doi: 10.1097/PCC.0b013e3181b806fc 19794327

[15] Rinchai D Anguiano E Nguyen P Chaussabel D. Finger Stick Blood Collection for Gene Expression Profiling and Storage of Tempus Blood RNA Tubes. F1000Res (2016) 5:1385. doi: 10.12688/f1000research.8841.1 28357036 PMC5357033

[16] Joint Genome Institute. A DOE Office of Science User Facility. BBDuk Guide. Available at: https://jgi.doe.gov/data-and-tools/bbtools/bb-tools-user-guide/bbduk-guide/ [Accessed February 2019]

[17] Genome Reference Consortium Human Build 38 Patch Release 9. Available at: http://www.ncbi.nlm.nih.gov/assembly/GCF_000001405.35/ [Accessed February 2019]

[18] Dobin A Davis CA Schlesinger F Drenkow J Zaleski C Jha S. STAR: Ultrafast Universal RNA-Seq Aligner. Bioinformatics (2013) 29(1):15–21. doi: 10.1093/bioinformatics/bts635 23104886 PMC3530905

[19] Walter and Eliza Hall Institute of Medical Research. Featurecounts: A Ultrafast and Accurate Read Summarizartion Program. Available at: http://bioinf.wehi.edu.au/featureCounts/ [Accessed February 2019]

[20] Love MI Huber W Anders S. Moderated Estimation of Fold Change and Dispersion for RNA-Seq Data With Deseq2. Genome Biol (2014) 15(12):550. doi: 10.1186/s13059-014-0550-8 25516281 PMC4302049

[21] Xue M Xie J Liu L Huang Y Guo F Xu J. Early and Dynamic Alterations of Th2/Th1 in Previously Immunocompetent Patients With Community-Acquired Severe Sepsis: A Prospective Observational Study. J Trans Med (2019) 17(1):57. doi: 10.1186/s12967-019-1811-9 PMC6391803 30813927

[22] Gupta DL Bhoi S Mohan T Galwnkar S Rao DN. Coexistence of Th1/Th2 and Th17/Treg Imbalances in Patients With Post Traumatic Sepsis. Cytokine (2016) 88:214–21. doi: 10.1016/j.cyto.2016.09.010 27676155

[23] Wu HP Chung K Lin CY Jiang BY Chuang DY Liu YC. Associations of T Helper 1, 2, 17 and Regulatory T Lymphocytes With Mortality in Severe Sepsis. Inflammation Res (2013) 62(8):751–63. doi: 10.1007/s00011-013-0630-3 PMC3712133 23670410

[24] Felmet KA Hall MW Clark RS Jaffe R Carcillo JA. Prolonged Lymphopenia, Lymphoid Depletion, and Hypoprolactinemia in Children With Nosocomial Sepsis and Multiple Organ Failure. J Immunol (2005) 174(6):3765–72. doi: 10.4049/jimmunol.174.6.3765 15749917

[25] Muszynski JA Nofziger R Greathouse K Steele L Hanson-Huber L Nateri J. Early Adaptive Immune Suppression in Children With Septic Shock: A Prospective Observational Study. Crit Care (2014) 18(4):R145. doi: 10.1186/cc13980 25005517 PMC4226962

[26] Monneret G Lepape A Voirin N Bohe J Venet F Debard AL. Persisting Low Monocyte Human Leukocyte Antigen-DR Expression Predicts Mortality in Septic Shock. Intensive Care Med (2006) 32(8):1175–83. doi: 10.1007/s00134-006-0204-8 16741700

[27] Landelle C Lepape A Voirin N Tognet E Venet F Bohe J. Low Monocyte Human Leukocyte Antigen-DR Is Independently Associated With Nosocomial Infections After Septic Shock. Intensive Care Med (2010) 36(11):1859–66. doi: 10.1007/s00134-010-1962-x 20652682

[28] Ravindran R Foley J Stoklasek T Glimcher LH McSorley SJ. Expression of T-Bet by CD4 T Cells is Essential for Resistance to Salmonella Infection. J Immunol (2005) 175(7):4603–10. doi: 10.4049/jimmunol.175.7.4603 16177105

[29] Szabo SJ Kim ST Costa GL Zhang X Fathman CG Glimcher LH. A Novel Transcription Factor, T-Bet, Directs Th1 Lineage Commitment. Cell (2000) 100(6):655–69. doi: 10.1016/S0092-8674(00)80702-3 10761931

[30] Sullivan BM Jobe O Lazarevic V Vasquez K Bronson R Glimcher LH. Increased Susceptibility of Mice Lacking T-Bet to Infection With Mycobacterium Tuberculosis Correlates With Increased IL-10 and Decreased IFN-Gamma Production. J Immunol (2005) 175(7):4593–602. doi: 10.4049/jimmunol.175.7.4593 16177104

[31] Szabo SJ Sullivan BM Stemmann C Satoskar AR Sleckman BP Glimcher LH. Distinct Effects of T-Bet in TH1 Lineage Commitment and IFN-Gamma Production in CD4 and CD8 T Cells. Science (2002) 295(5553):338–42. doi: 10.1126/science.1065543 11786644

[32] Sullivan BM Juedes A Szabo SJ von Herrath M Glimcher LH. Antigen-Driven Effector CD8 T Cell Function Regulated by T-Bet. Proc Natl Acad Sci U.S.A. (2003) 100(26):15818–23. doi: 10.1073/pnas.2636938100 PMC307651 14673093

[33] Intlekofer AM Banerjee A Takemoto N Gordon SM Dejong CS Shin H. Anomalous Type 17 Response to Viral Infection by CD8+ T Cells Lacking T-Bet and Eomesodermin. Science (2008) 321(5887):408–11. doi: 10.1126/science.1159806 PMC2807624 18635804

[34] Cruz-Guilloty F Pipkin ME Djuretic IM Levanon D Lotem J Lichtenheld MG. Runx3 and T-Box Proteins Cooperate to Establish the Transcriptional Program of Effector CTLs. J Exp Med (2009) 206(1):51–9. doi: 10.1084/jem.20081242 PMC2626671 19139168

[35] Pipkin ME Sacks JA Cruz-Guilloty F Lichtenheld MG Bevan MJ Rao A. Interleukin-2 and Inflammation Induce Distinct Transcriptional Programs That Promote the Differentiation of Effector Cytolytic T Cells. Immunity (2010) 32(1):79–90. doi: 10.1016/j.immuni.2009.11.012 20096607 PMC2906224

[36] Intlekofer AM Takemoto N Wherry EJ Longworth SA Northrup JT Palanivel VR. Effector and Memory CD8+ T Cell Fate Coupled by T-Bet and Eomesodermin. Nat Immunol (2005) 6(12):1236–44. doi: 10.1038/ni1268 16273099

[37] Romero V Andrade F. Non-Apoptotic Functions of Granzymes. Tissue Antigens (2008) 71(5):409–16. doi: 10.1111/j.1399-0039.2008.01013.x 18331532

[38] Smyth MJ Kelly JM Sutton VR Davis JE Browne KA Sayers TJ. Unlocking the Secrets of Cytotoxic Granule Proteins. J Leukoc Biol (2001) 70(1):18–29. doi: 10.1189/jlb.70.1.18 11435481

[39] Wang L Li Q Wu L Liu S Zhang Y Yang X. Identification of SERPINB1 as a Physiological Inhibitor of Human Granzyme H. J Immunol (2013) 190(3):1319–30. doi: 10.4049/jimmunol.1202542 23269243

[40] Palmer C Diehn M Alizadeh AA Brown PO. Cell-Type Specific Gene Expression Profiles of Leukocytes in Human Peripheral Blood. BMC Genomics (2006) 7:115. doi: 10.1186/1471-2164-7-115 16704732 PMC1479811

[41] van Domselaar R Quadir R van der Made AM Broekhuizen R Bovenschen N. All Human Granzymes Target hnRNP K That Is Essential for Tumor Cell Viability. J Biol Chem (2012) 287(27):22854–64. doi: 10.1074/jbc.M112.365692 PMC3391115 22582387

[42] Gordon SM Chaix J Rupp LJ Wu J Madera S Sun JC. The Transcription Factors T-Bet and Eomes Control Key Checkpoints of Natural Killer Cell Maturation. Immunity (2012) 36(1):55–67. doi: 10.1016/j.immuni.2011.11.016 22261438 PMC3381976

[43] Townsend MJ Weinmann AS Matsuda JL Salomon R Farnham PJ Biron CA. T-Bet Regulates the Terminal Maturation and Homeostasis of NK and Valpha14i NKT Cells. Immunity (2004) 20(4):477–94. doi: 10.1016/S1074-7613(04)00076-7 15084276

[44] Werneck MB Lugo-Villarino G Hwang ES Cantor H Glimcher LH. T-Bet Plays a Key Role in NK-Mediated Control of Melanoma Metastatic Disease. J Immunol (2008) 180(12):8004–10. doi: 10.4049/jimmunol.180.12.8004 PMC3709580 18523263

[45] Shakoory B Carcillo JA Chatham WW Amdur RL Zhao H Dinarello CA. Interleukin-1 Receptor Blockade Is Associated With Reduced Mortality in Sepsis Patients With Features of Macrophage Activation Syndrome: Reanalysis of a Prior Phase III Trial. Crit Care Med (2016) 44(2):275–81. doi: 10.1097/CCM.0000000000001402 PMC5378312 26584195

[46] Kokkotou E Torres D Moss AC O’Brien M Grigoriadis DE Karalis K. Corticotropin-Releasing Hormone Receptor 2-Deficient Mice Have Reduced Intestinal Inflammatory Responses. J Immunol (2006) 177(5):3355–61. doi: 10.4049/jimmunol.177.5.3355 16920976

[47] Baigent SM. Peripheral Corticotropin-Releasing Hormone and Urocortin in the Control of the Immune Response. Peptides (2001) 22(5):809–20. doi: 10.1016/S0196-9781(01)00395-3 11337095

[48] Kim BJ Kayembe K Simecka JW Pulse M Jones HP. Corticotropin-Releasing Hormone Receptor-1 and 2 Activity Produces Divergent Resistance Against Stress-Induced Pulmonary Streptococcus Pneumoniae Infection. J neuroimmunology (2011) 237(1-2):57–65. doi: 10.1016/j.jneuroim.2011.06.016 21774994 PMC5715473

[49] Harle G Kaminski S Dubayle D Frippiat JP Ropars A. Murine Splenic B Cells Express Corticotropin-Releasing Hormone Receptor 2 That Affect Their Viability During a Stress Response. Sci Rep (2018) 8(1):143. doi: 10.1038/s41598-017-18401-y 29317694 PMC5760685

[50] Sun L Ye RD. Role of G Protein-Coupled Receptors in Inflammation. Acta Pharmacol Sin (2012) 33(3):342–50. doi: 10.1038/aps.2011.200 PMC4085652 22367283

[51] Vermette D Hu P Canarie MF Funaro M Glover J Pierce RW. Tight Junction Structure, Function, and Assessment in the Critically Ill: A Systematic Review. Intensive Care Med Exp (2018) 6(1):37. doi: 10.1186/s40635-018-0203-4 30259344 PMC6158145

[52] Atkinson SJ Varisco BM Sandquist M Daly MN Klingbeil L Kuethe JW. Matrix Metalloproteinase-8 Augments Bacterial Clearance in a Juvenile Sepsis Model. Mol Med (2016) 22:455–63. doi: 10.2119/molmed.2016.00058 PMC5072408 27506554

[53] Lauhio A Hastbacka J Pettila V Tervahartiala T Karlsson S Varpula T. Serum MMP-8, -9 and TIMP-1 in Sepsis: High Serum Levels of MMP-8 and TIMP-1 Are Associated With Fatal Outcome in a Multicentre, Prospective Cohort Study. Hypothetical impact tetracyclines. Pharmacol Res (2011) 64(6):590–4. doi: 10.1016/j.phrs.2011.06.019 21742038

[54] Garraud O Cognasse F. Are Platelets Cells? And If Yes, are They Immune Cells? Front Immunol (2015) 6:70. doi: 10.3389/fimmu.2015.00070 25750642 PMC4335469

