# Supplementary figures and images for: Transcriptomic Profiles in Children With Septic Shock With or Without Immunoparalysis
Source: Front Immunol. 2021 Oct 1;12:733834. doi: 10.3389/fimmu.2021.733834 (PMC8517409; doi:10.3389/fimmu.2021.733834)

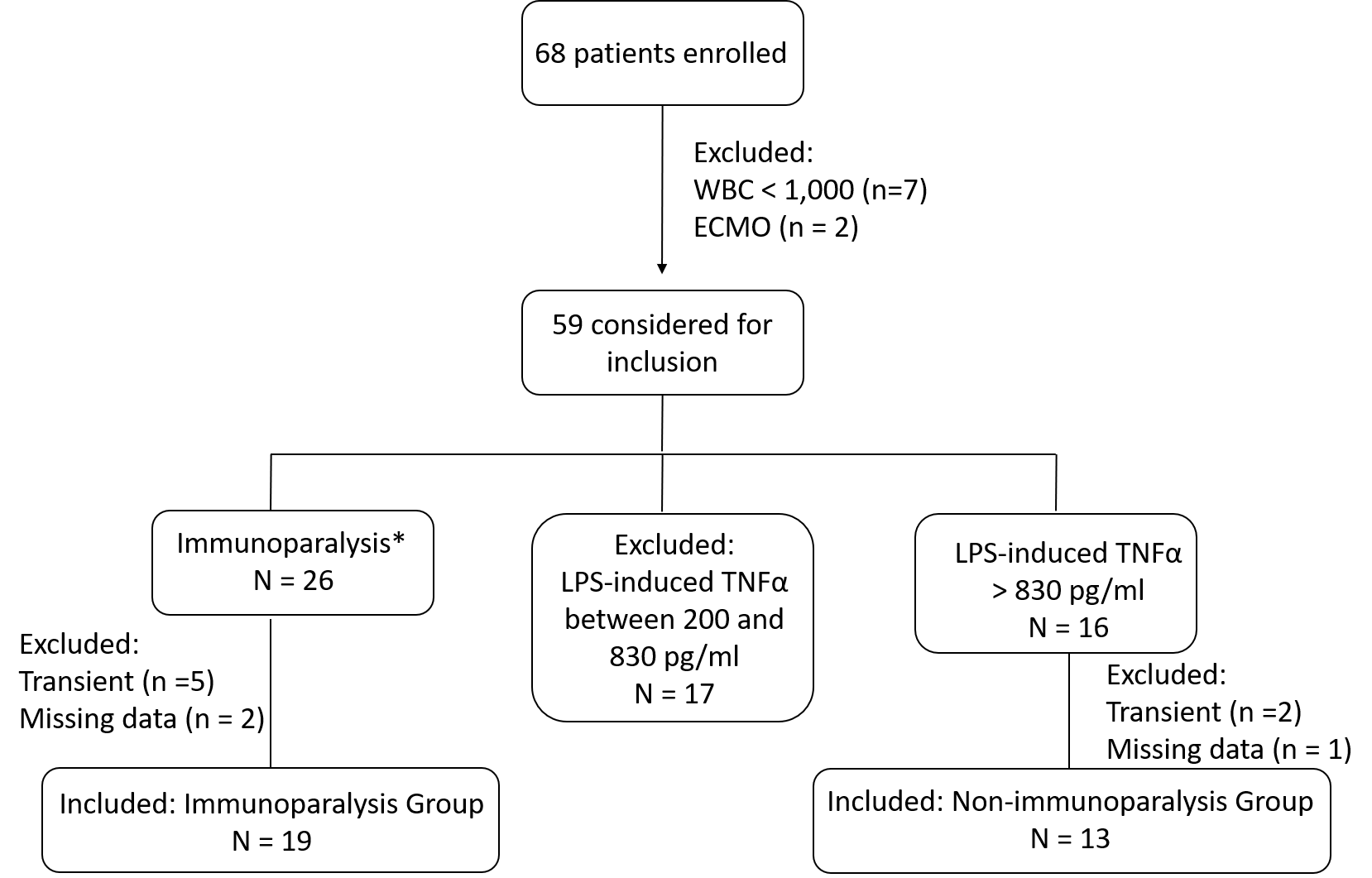

Supplement: Supplementary Figure 1 — Consort diagram depicting patient inclusion and exclusion from analyses and allocation into groups. *Immunoparalysis is defined as ex vivo LPS-induced TNFα response < 200 pg/ml, per our previous publications. [file Image_1.tif]

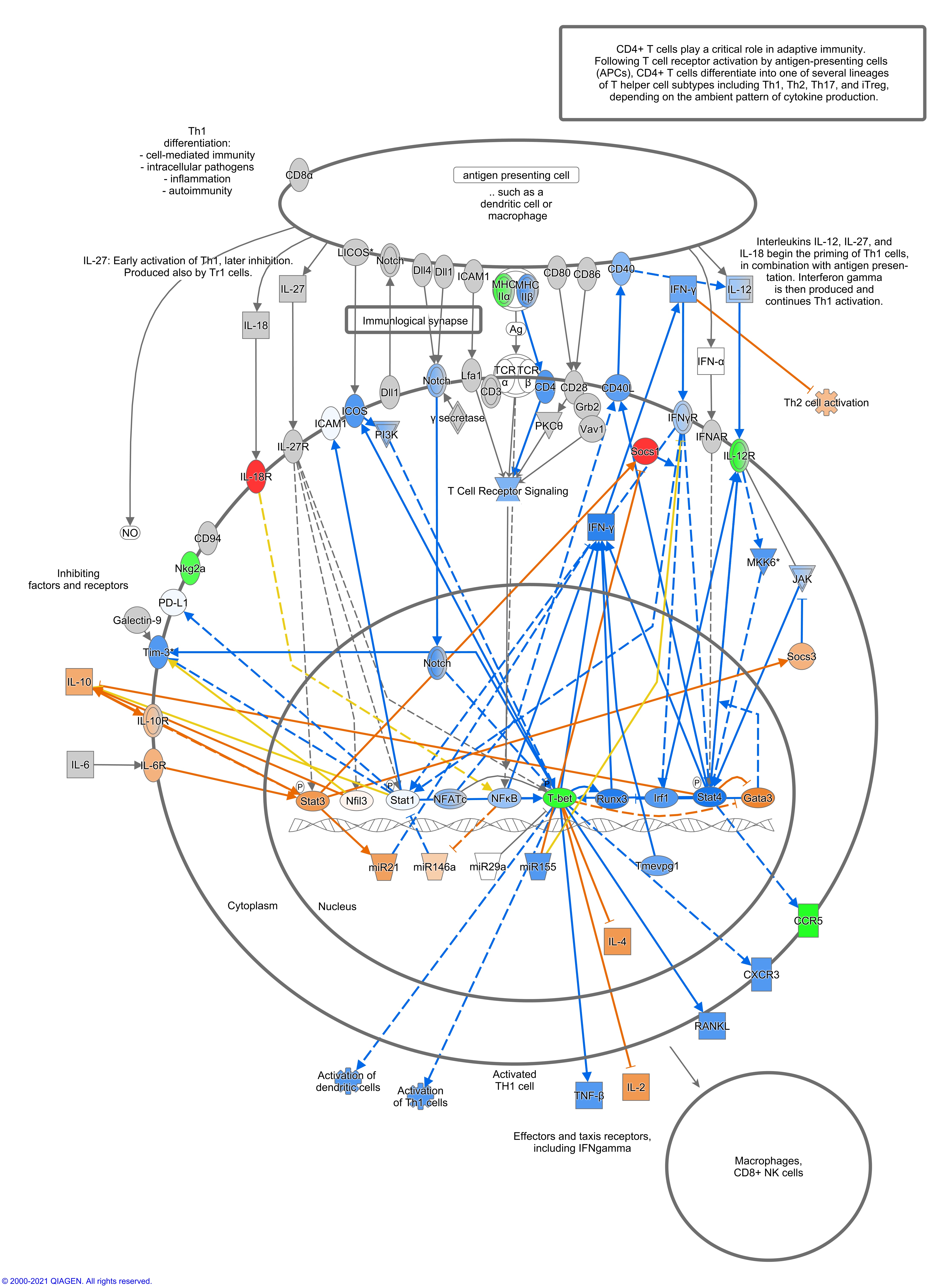

Supplement: Supplementary Figure 2 — Figure depicting molecules in the Th1 Pathway. Green color represents molecules that are downregulated in the IPA dataset. Red color represents molecules that are upregulated in the IPA dataset. Blue color represents molecules that are predicted to be inhibited based on the IPA dataset. Orange color represents molecules that are predicted to be activated based on the IPA dataset. [file Image_2.jpeg]

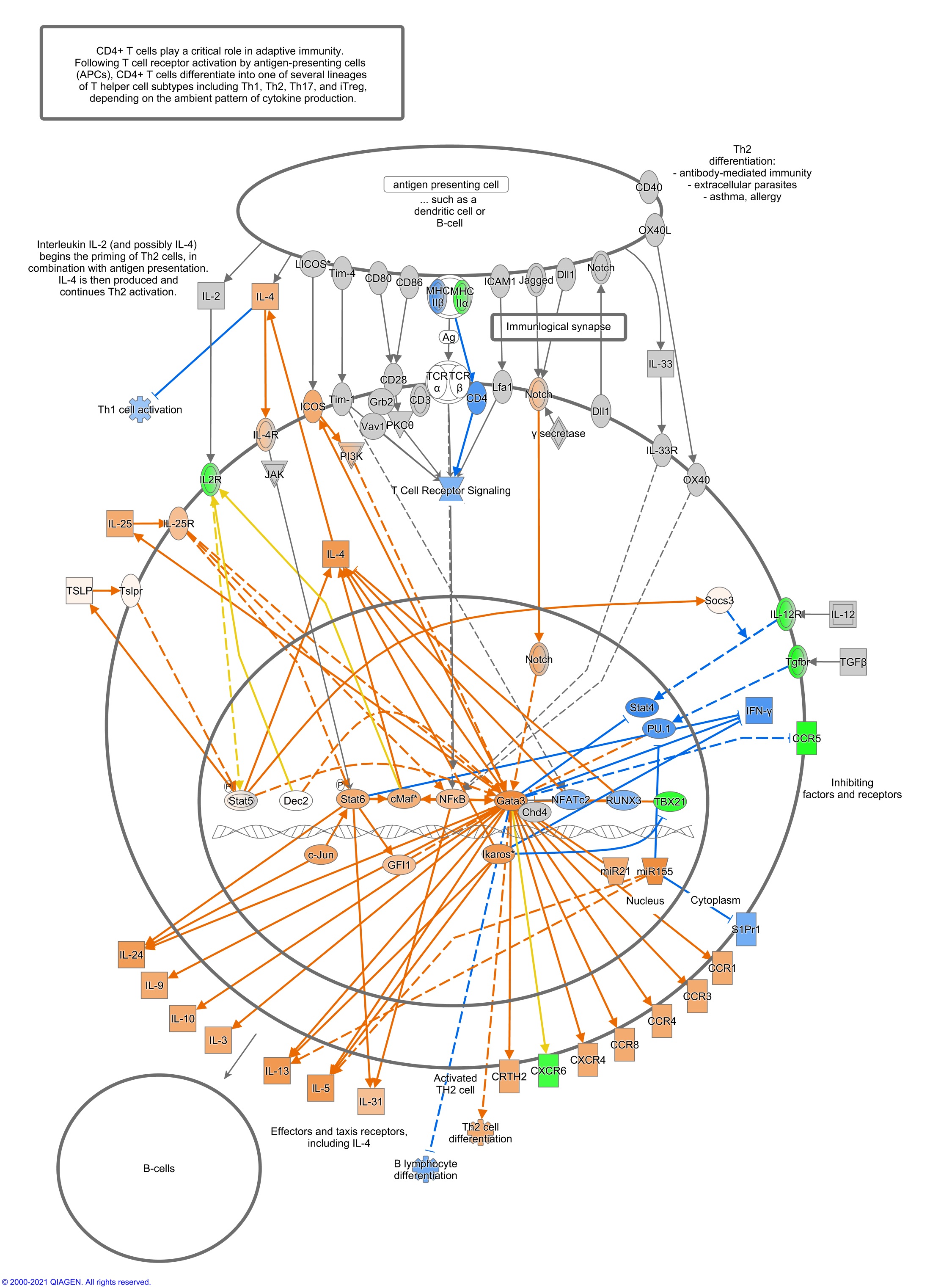

Supplement: Supplementary Figure 3 — Figure depicting molecules in the Th2 Pathway. Green color represents molecules that are downregulated in the IPA dataset. Red color represents molecules that are upregulated in the IPA dataset. Blue color represents molecules that are predicted to be inhibited based on the IPA dataset. Orange color represents molecules that are predicted to be activated based on the IPA dataset. [file Image_3.jpeg]

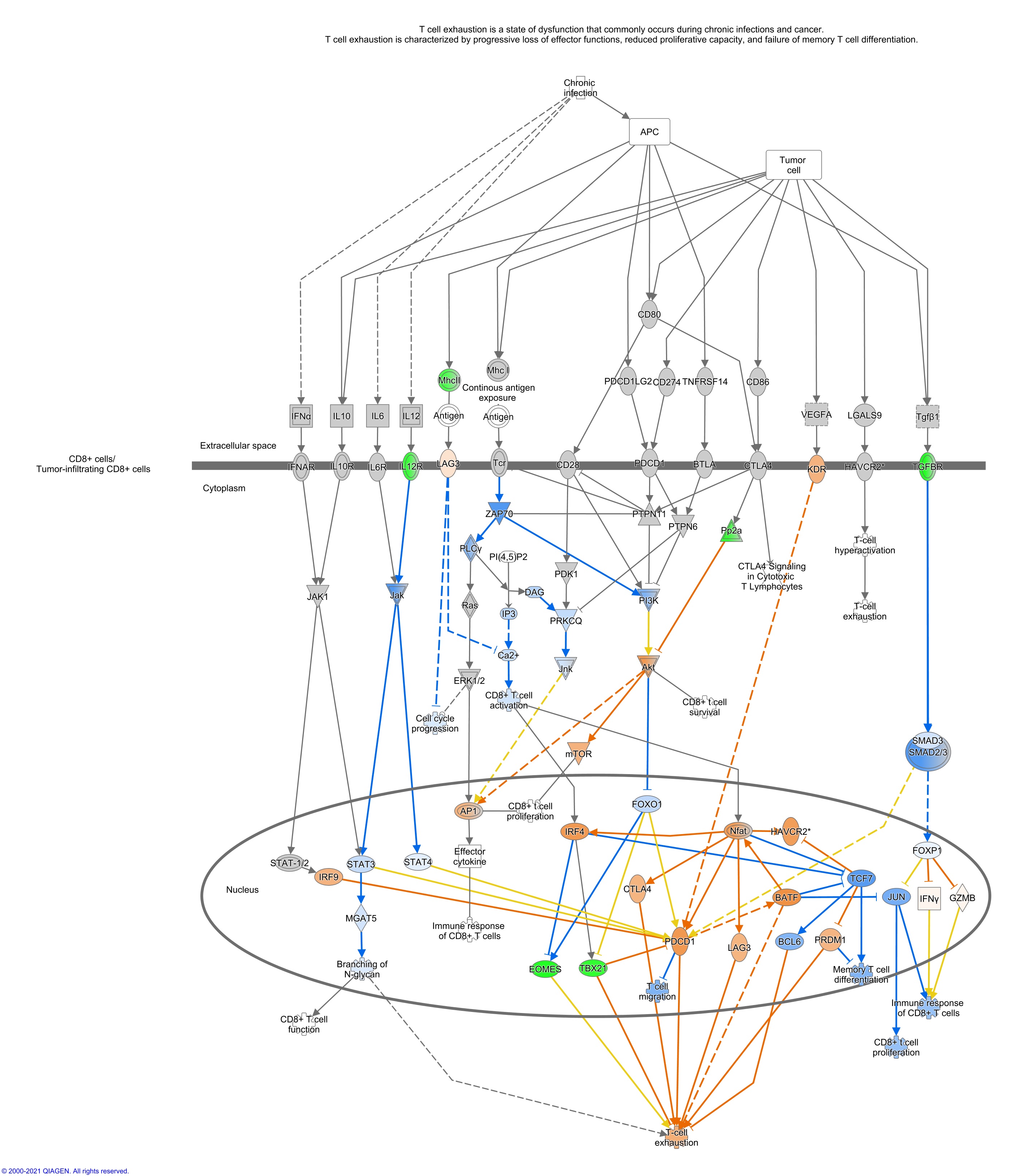

Supplement: Supplementary Figure 4 — Figure depicting molecules in the T cell Exhaustion Pathway. Green color represents molecules that are downregulated in the IPA dataset. Red color represents molecules that are upregulated in the IPA dataset. Blue color represents molecules that are predicted to be inhibited based on the IPA dataset. Orange color represents molecules that are predicted to be activated based on the IPA dataset. [file Image_4.jpeg]

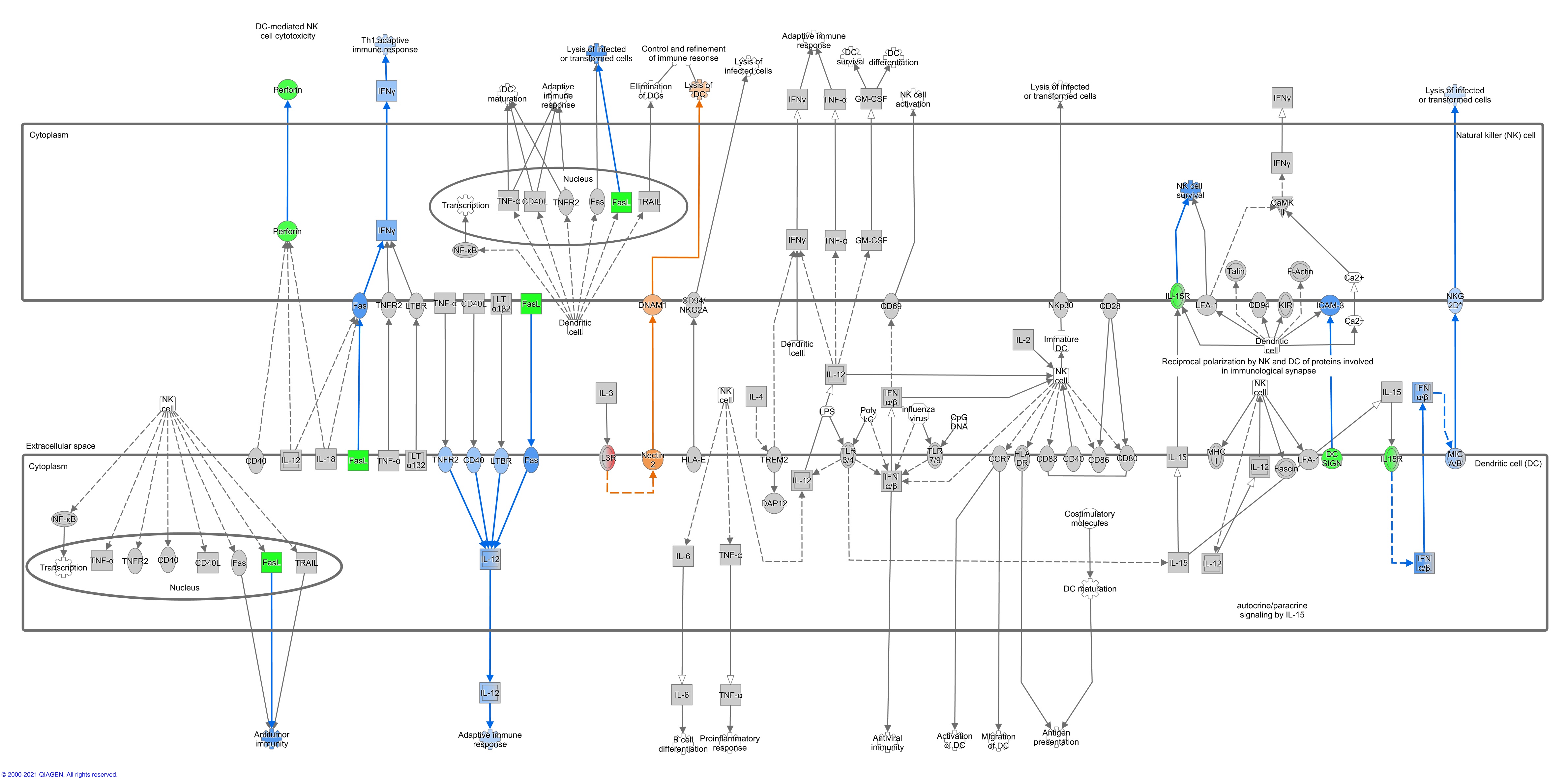

Supplement: Supplementary Figure 5 — Figure depicting molecules in the Crosstalk between Dendritic Cells and Natural Killer Cells Pathway. Green color represents molecules that are downregulated in the IPA dataset. Red color represents molecules that are upregulated in the IPA dataset. Blue color represents molecules that are predicted to be inhibited based on the IPA dataset. Orange color represents molecules that are predicted to be activated based on the IPA dataset. [file Image_5.jpeg]
